# Stabilization and formulation of a recombinant Human Cytomegalovirus vector for use as a candidate HIV-1 vaccine

**DOI:** 10.1016/j.vaccine.2019.09.027

**Published:** 2019-10-16

**Authors:** Ozan S. Kumru, Soraia Saleh-Birdjandi, Lorena R. Antunez, Eddy Sayeed, David Robinson, Sjoerd van den Worm, Geoffrey S. Diemer, Wilma Perez, Patrizia Caposio, Klaus Früh, Sangeeta B. Joshi, David B. Volkin

**Affiliations:** a Department of Pharmaceutical Chemistry, Vaccine Analytics and Formulation Center, University of Kansas, Lawrence, KS 66047, USA; b International AIDS Vaccine Initiative, 125 Broad Street, 9th Floor, New York, NY 10004, USA; c Robinson Vaccines and Biologics LLC, New York, NY, USA; d Oregon Health & Science University, Vaccine and Gene Therapy Institute, 505 NW185th Ave, Beaverton, OR 97006, USA

**Keywords:** HIV vaccine, Cytomegalovirus, Formulation, Freeze-thaw, Stability, Excipient, BDS, Bulk Drug Substance, BSA, bovine serum albumin, CMV, cytomegalovirus, CO_2_, carbon dioxide, cP, centipoise, DAPI, 4′,6-diamidino-2-phenylindole, DTT, dithiothreitol, dPBS, Dulbecco's phosphate-buffered salin*e*, FBS, fetal bovine serum, FFU, fluorescence focus units, FITC, fluorescein isothiocyanate, HNS buffer, 25 mM Histidine, 150 mM NaCl, 10% (w/v) sucrose, pH 6.0, IE-IFA, intermediate-early indirect immunofluorescence assay, Log, log units are in base 10, MOI, multiplicity of infection, OHSU, Oregon Health Sciences University, PDL, population doubling level, PBS, phosphate buffered saline, pH 7.4, PP, polypropylene, PVDF, polyvinylidene difluoride, TNS buffer, 50 mM Tris, 150 mM NaCl, 10% (w/v) sucrose, pH 8.0, w/v, weight/volume

## Abstract

Live attenuated viral vaccine/vector candidates are inherently unstable and infectivity titer losses can readily occur without defining appropriate formulations, storage conditions and clinical handling practices. During initial process development of a candidate vaccine against HIV-1 using a recombinant Human Cytomegalovirus vector (rHCMV-1), large vector titer losses were observed after storage at 4 °C and after undergoing freeze-thaw. Thus, the goal of this work was to develop candidate frozen liquid formulations of rHCMV-1 with improved freeze-thaw and short-term liquid stability for potential use in early clinical trials. To this end, a virus stability screening protocol was developed including use of a rapid, *in vitro* cell-based immunofluorescence focus assay to quantitate viral titers. A library of ∼50 pharmaceutical excipients (from various known classes of additives) were evaluated for their effect on vector stability after freeze-thaw cycling or incubation at 4 °C for several days. Certain additives including sugars and polymers (e.g., trehalose, sucrose, sorbitol, hydrolyzed gelatin, dextran 40) as well as removal of NaCl (lower ionic strength) protected rHCMV-1 against freeze-thaw mediated losses in viral titers. Optimized solution conditions (e.g., solution pH, buffers and sugar type) slowed the rate of rHCMV-1 titer losses in the liquid state at 4 °C. After evaluating various excipient combinations, three new candidate formulations were designed and rHCMV-1 stability was benchmarked against both the currently-used and a previously reported formulation. The new candidate formulations were significantly more stable in terms of reducing rHCMV-1 titer losses after 5 freeze-thaw cycles or incubation at 4 °C for 30 days. This case study highlights the utility of semi-empirical design of frozen liquid formulations of a live viral vaccine candidate, where protection against infectivity titer losses due to freeze-thaw and short-term liquid storage are sufficient to enable more rapid initiation of early clinical trials.

## 1 Introduction

Human Cytomegaloviruses (HCMV) are enveloped, dsDNA viruses classified as members of the family *Herpesviridae* [1]. The HCMV genome is ∼235 kb in length, encodes ∼165 open reading frames [2], and assembled virions are 150–200 nm in diameter [3]. Vaccination using recombinant rhesus Cytomegalovirus (RhCMV) vectors (containing the appropriate inserts) have shown promising results in conferring protection in non-human primates against SIV, *Mycobacterium tuberculosis*, and malaria (*Plasmodium knowlesi*) infections [4], [5], [6]. Moreover, since recombinant CMV vectors can stimulate production of high concentrations of systemic effector memory T-cells, appropriately designed vectors have the potential to clear SIV/HIV and *Mycobacterium tuberculosis* infections [4], [5], [7], [8]. Importantly, live attenuated RhCMV maintained this unique long-lived immunogenicity and protection against SIV despite being spread-deficient [9], [10]. Such promising preclinical animal results have generated much interest in performing initial clinical trials of a HCMV-based HIV vaccine candidate. Although recombinant HCMV expressing heterologous antigens has never been clinically tested, several attenuated HCMV vaccine candidates have entered early clinical trials as vaccines against HCMV [11], [12]. Nevertheless, there are no licensed CMV vaccines, and there are limited publications on their pharmaceutical development.

During early process development of a small-scale production process to generate initial clinical material, it was observed that HCMV-based vectors were prone to accelerated titer loss upon freeze-thaw and/or liquid storage (at 2–8 °C or 25 °C; unpublished data). Since vector titer losses during a manufacturing process reduces overall yields and increases production costs, it is highly desirable to improve virus stability by minimizing exposure to stresses that cause inactivation and by optimizing solution conditions. In general, live viral vaccine/vectors are inherently unstable from a pharmaceutical perspective, particularly upon exposure to elevated temperatures and/or multiple freeze-thaw cycles [13], [14]. These stresses, along with others commonly encountered during bulk and drug product manufacturing (e.g., agitation, adsorption), can lead to unacceptable losses in virus titers during production and long-term storage [15], [16]. Furthermore, infectivity titer losses can also occur during administration of live viruses to patients if appropriate handling protocols are not carefully defined and followed [14], [17]. Thus, to facilitate first-in-human clinical trials, development of a frozen liquid formulation that provides sufficient vector stability during frozen storage, freeze-thaw and transient storage in liquid state is required [18], [19].

The goal of this work was to identify conditions to stabilize a live-attenuated recombinant human cytomegalovirus vaccine vector that encodes HIV-gag (rHCMV-1) against freeze-thaw and short-term storage at 4 °C for potential use in initial Phase 1 clinical trials as a vaccine candidate against HIV-1. We evaluated various classes and types of pharmaceutical excipients (∼50 total) for their ability to stabilize rHCMV-1 versus these stresses. First, we developed a virus stability screening protocol that utilized an *in vitro* cell-based immunofluorescence focus assay (to quantitate viral titers) with shorter assay incubation times and thus greatly improved sample throughput. Various combinations of promising stabilizers were then evaluated and the stability profile of rHCMV-1 in three candidate formulations were benchmarked against both the currently used and a previously reported CMV vaccine formulation. These results are discussed not only in terms of identifying candidate formulations resulting in improved rHCMV-1 stability, but also to better understand the possible causes and mechanisms of rHCMV-1 titer losses that can occur during manufacturing, long-term storage and administration to patients.

## 2 Materials and methods

### 2.1 Generation of rHCMV-1

The HCMV-TR3 vector backbone (Genbank accession number MN075802) is a modified version of the cloned clinical isolate HCMV TR [20] (GenBank Accession Number: AC146906.1). The construction, *in vitro* and *in vivo* characterization of HCMV-TR3 and HCMV-TR3-derived vectors will be published elsewhere [21]. HCMV-TR3 was live-attenuated by replacing the pp71-encoding gene UL82 with HIVgag. UL82 is required for growth *in vitro* at low MOI [22]. HIVgag was derived from the previously described GRIN-plasmid [23] provided by IAVI. Additionally, the vector was tropism restricted by deleting the genes UL128 and UL130 which are subunits of a pentameric complex required for infection of epithelial, endothelial and monocytic cells [24]. A bacterial artificial chromosome (BAC) of the resulting recombinant HCMV TR3ΔUL82gagΔUL128-130 was used to generate IND*Ready* DNA preparations using dedicated fermentation vessels, tubing, mixing vessels, and using animal-free products or treated in compliance with appropriate regulations to reduce the risk of TSE/BSE (Puresyn Inc.). IND*Ready* BAC-DNA was electroporated into MRC-5 cells (provided by IAVI) and the live-attenuated vector was reconstituted in a T-flask in DMEM containing 10% fetal bovine serum (FBS), qualified, Australian origin, and 2 mM GlutaMAX (ThermoFisher) in the presence of siRNA targeting the host cell factor DAXX to support growth of pp71-deficient HCMV [25]. The vector was cloned by limiting dilution in 96-well plates containing DAXX siRNA-transfected MRC-5 cells that were infected with virus diluted to 3, 1, or 0.3 FFU/well. After 21–28 days in culture, the 96-well plates were examined for the number of wells containing a single focus of infection. According to the Poisson distribution for clonal purification, we used plates containing <10 positive wells to expand clones from individual wells. Clone 5N2F9 was selected for further expansion and characterization. HCMV TR3ΔUL82gagΔUL128-130 clone 5N2F9 is referred to as rHCMV-1.

To generate a viral seed stock, we expanded rHCMV-1 initially in a 48-well plate (passage 1), followed by expansion into a T25 flask, two T150 flasks, and finally 30 T150 flasks (passages 2, 3, and 4, respectively). The clonal virus was then used to infect DAXX-siRNA transfected MRC-5 cells in a 12-layer HYPERstack (HS-12) (Corning) and after a second DAXX siRNA transfection at 12 days post-infection, the media was changed to DMEM containing 0.2% FBS and 2 mM GlutaMAX. Upon achieving full cytopathic effect (CPE) the supernatant was harvested and virus was clarified by centrifugation at 4000*g* for 15 min at 4 °C to remove large cell debris, then purified by ultrafiltration and diafiltration. The clarified virus supernatant was first concentrated 20-fold by tangential flow filtration (TFF) using a Repligen (Waltham, Massachusetts) MiniKros 1000 cm^2^ polysulfone hollow-fiber TFF column with a pore diameter of 50 nm and interior fiber diameter of 0.5 mm. Immediately following concentration, the virus culture supernatant was exchanged with 12.5 concentrate volumes of TNS buffer (50 mM Tris, 150 mM NaCl, 10% sucrose, pH 8.0) by diafiltration in the TFF column. A shear rate of 2400 s^−1^ was used for both TFF and diafiltration processes, maintaining an average TMP of 1.5 psi. The titer was determined on pp71-complementing cells by fluorescence focus assay at 9.22 ± 0.97E6 FFU/mL. The material was characterized for sterility (mycoplasma), genetic identity (next generation sequencing of the entire genome and qPCR for gene deletions) as well as antigen expression (immunoblot for HIVgag).

Aliquots of virus were stored at −80 °C in polypropylene cryogenic tubes, thawed at room temperature, and kept on ice prior to dilution into a given formulation as described below. Excipients were purchased from Sigma-Aldrich (St. Louis, MO) except for sucrose, trehalose, and mannitol (purchased from Pfanstiehl Inc., Waukegan, IL) and sulfobutylether-β-cyclodextrin (provided by Ligand Pharmaceuticals, Lawrence, KS). All excipients were of high purity (≥99%).

### 2.2 Cell culture conditions for viral potency assay

Adherent pp71-complementing cells (BJ-5ta pp71-rtTA) were provided by OHSU and cultured in BJpp71 medium, which was prepared by mixing 400 mL of DMEM (Life Technologies, Carlsbad, CA), 100 mL Media 199 (Corning Cellgrow, Tewksbury, MA), 50 mL heat-inactivated tetracycline free FBS (Atlanta Biologicals), 5 mL 100X Pen-Strep-Glutamate, 100 µg/mL G418 Hygromycin, and 5 µg/mL Blasticidin (purchased from Life Technologies, Carlsbad, CA). The BJ-5ta pp71-rtTA cells constitutively express the TetOn transactivator and the HCMV pp71 protein under the control of the doxycycline inducible Tet promoter [21]. Cells were cultured in T150 flasks (Corning Tewksbury, MA) in a humidified 37 °C, 5% CO_2_ incubator. Cells were passaged at least twice per week when the flasks were at least 90% confluent and the population doubling level (PDL) was maintained ≤40.

### 2.3 Immediate-early fluorescence focus assay (IE-FFA)

BJ-5ta pp71-rtTA cells were harvested from a confluent culture and seeded in a 96 well plate (Thermo-Scientific Nunc Edge 96-Well) at 12,000 cells/well (in 0.1 mL total volume) in BJpp71 medium supplemented with 1 µg/mL doxycycline (Clonetech, Mountain View, CA) and incubated at 37 °C, 5% CO_2_ for ∼24 h. The media was exchanged with 70 µl of complete media supplemented with 1 µg/mL doxycycline, cells were infected with 30 µl of virus and incubated at 37 °C, 5% CO_2_ for ∼18 h. The culture medium was removed, cells were fixed with 0.1 mL of 4% formaldehyde (in dPBS, methanol-free, Pierce) for 10 min, followed by two washes of 0.15 mL of dPBS. Cells were subsequently quenched in 0.1 mL of 50 mM ammonium chloride (Thermo-Fisher, Waltham, MA) for 10 min followed by two washes of 0.15 mL of dPBS. The cells were then permeabilized in 0.1 mL of 0.1% (w/v) Triton X-100 (VWR, Radnor, PA) for 5 min, followed by three washes in 0.15 mL of dPBS with 2% (w/v) BSA (Sigma, St. Louis, MO). The liquid was removed and cells were stained with 50 µl of anti-IE72 antibody conjugated to AlexaFluor 488, Clone:81B1.2 (EMB Millipore # MAB810X, Burlington, MA) that was diluted 1:100 in dPBS containing 2% BSA and incubated at 37 °C for one hour followed by three washes with 0.15 mL of dPBS containing 2% BSA, and one final wash of 0.15 mL dPBS for 10 min each in the dark. The plate was inverted and allowed to dry in the dark. Fifty microliters of DAPI-Fluoromount-G clear mounting media (Southern Biotech, Birmingham, AL) was added to each well and foci were quantified by fluorescence microscopy.

### 2.4 Fluorescence microscopy, virus titer calculation, and log loss viral stability calculations

The 96 well plates were loaded in the plate holder of a Nikon Eclipse Ti-E fluorescence (Nikon, Melville, NY) microscope equipped with an automated stage and a LED light source. The plates were covered with aluminum foil and ambient lights were switched off during imaging. A custom macro was designed and executed for foci quantitation in automatic mode using 4X magnification, 50% LED intensity, and exposure time on the FITC and DAPI channels set to auto. The microscope was programmed to autofocus on the FITC signal in each well with a range of 100 µm and step size of 20 µm on a single pass. The FITC filter was used to visualize the AlexaFluor 488 signal (which designates a virus infected cell) and the DAPI filter was used to visualize the cell nuclei (to count total cell number, both infected and uninfected). Plates were read top to bottom, reading a single point in the center of each well. Foci were counted by bright spot detection of a circular area using a typical diameter of 14.6 µm and a contrast of ∼4.095 (DAPI) and ∼36.85 (FITC). For the DAPI foci, a smoothing function was applied under the binary processing function. Dark background was subtracted at a power of 1, and for the FITC signal, local contrast was applied at a size of 20 and a power of 50%.

The resulting fluorescence foci counts (FITC, representing virus infected cells vs. DAPI, which represents the total number of cells) obtained from the microscope were applied to the following equations to calculate the virus titer.

1) *moi_calculated_ = -*ln *((1* − *percentage infected cells)/100)*
2) *titer_calculated_ = moi_calculated_ × cells/well/volume of virus used*

The percentage of infected cells was determined by the ratio of the FITC counts to the DAPI counts and the log loss of viral titer was calculated as follows:

$$\mathrm{Log}\;loss=Log\;titer\;\left(-80{\;}^{{}^{\circ}}C\;control\right)-Log\;titer\;\left(stressed\;sample\right)$$

The standard deviation (SD) of potency loss was calculated to account for error propagation in the control and stressed samples:

$$\mathrm{Standard}\;deviation=\sqrt{x^{2}+y^{2}}$$

where x is the SD of the −80 °C control and y is the SD of the experimental sample, respectively. The −80 °C control sample was compounded in parallel to the experimental samples and frozen at −80 °C in either TNS buffer or the respective formulation as specified in the text.

### 2.5 rHCMV-1 excipient screening studies

Excipient stock solutions were prepared by dissolving additives in ultrapure water (18.2 MΩ), pH adjusted using HCl or NaOH, and sterile filtered using a 0.22 µm PVDF filter (EMD Millipore, Burlington, MA). The rHCMV-1 vector was diluted at 1:100 or 1:150 into a formulation solution at 0.5 mL total volume. The sterile containers were either 1.5 mL polypropylene (PP) microcentrifuge tubes (Fisher-Scientific) or 2 mL glass vials (Fiolax clear type, Schott, Lebanon, PA) with FluroTec® coated 13 mm pre-sterilized stoppers (West Pharmaceuticals, West Whiteland Township, PA). For initial freeze-thaw excipient screening studies, samples were frozen at −80 °C for at least 24 h, thawed at room temperature until visually complete, and analyzed by IE-IFA. Unless otherwise specified, liquid stability samples (at 4 or 25 °C) were stored in stability chambers (Torrey Pines Scientific, Carlsbad, CA), removed at various timepoints, and analyzed by IE-IFA without freezing.

### 2.6 Real-time, accelerated, and freeze-thaw stability studies of the candidate formulations

Candidate formulations were compounded by diluting the rHCMV-1 stock to log (titer) = 4.8 FFU/mL (1:100 dilution into each candidate formulation with total volume of 0.5 mL), and filled into stoppered glass vials (see above). For liquid stability studies, samples were stored (4 or 25 °C for various times) and then frozen (−80 °C) prior to IE-IFA analysis. Unstressed control samples were frozen at −80 °C and analyzed together with stability samples as part of each IE-IFA assay to calculate the initial titer for the log loss calculations. For the freeze-thaw studies, samples were compounded as above, then freeze-thaw stressed (−20 or −80 °C) for 5 cycles by freezing for at least 24 h, followed by thawing at room temperature until visually thawed. Samples were stored in a −20 °C freezer lacking auto-defrost cycling.

## 3 Results

### 3.1 rHCMV-1 instability after multiple freeze-thaw cycles and short-term 4 °C storage

**Fig. 1:**
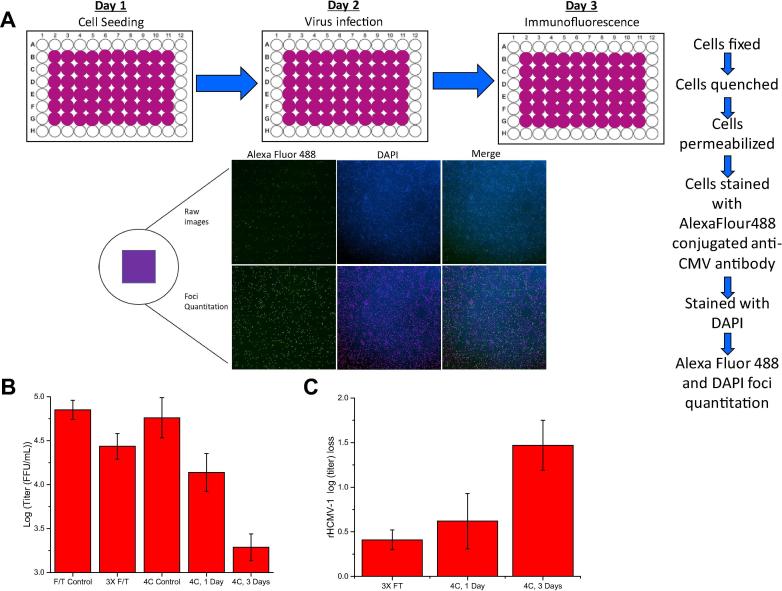
Overview of viral infectivity assay (immediate-early fluorescence focus assay, IE-IFA) and rHCMV-1 titer losses after freeze-thaw cycling and short-term storage at 4 °C in TNS buffer. (A) Schematic of the IE-IFA assay including immunofluorescence readout to determine number of infectious viral particles. (B) log (titer) values of rHCMV-1 control and stressed samples in TNS buffer (see text), and (C) log loss of rHCMV-1 viral titer (compared to control) after three freeze-thaw cycles from −80 °C or incubation at 4 °C for 1 or 3 days. Log loss was calculated by subtracting the log (titer) value of the control sample (viral vector stock in TNS buffer diluted 1:150 in TNS buffer and stored at −80 °C) from the log (titer) value of the stressed sample prepared in same manner. Stability data are an average of 12 measurements, except for the 4 °C, 3 day timepoint which was an average of 6 measurements, with the error bars representing the standard deviation.

The rHCMV-1 vector preparation was supplied in TNS buffer (50 mM Tris, 150 mM NaCl, 10% Sucrose, pH 8.0). As a first step in developing new candidate formulations with improved stability, the degradation profile of rHCMV-1 viral titer was established after multiple freeze-thaw cycles and after storage at 4 °C for 1–3 days in TNS buffer as measured by an *in vitro* cell-based immediate-early fluorescence focus assay (IE-IFA). The IE-IFA assay greatly facilitated the formulation development work described in this study by rapidly measuring the number of infectious virus particles in a 96-well plate format. As shown in Fig. 1A, after ∼18 h of virus infection, the cells were permeabilized, and an anti-IE72 antibody conjugated to AlexaFluor-488 were used to probe for number of infected cells (and compared total number of cells by DAPI staining). Titer values of rHCMV-1 samples are displayed in Fig. 1B, and loss of titer (i.e., log loss) was calculated by subtraction of the control samples from the stressed virus samples (Fig. 1C). After one and three freeze-thaw cycles, ∼0.0 and ∼0.40 log loss of titer was observed, respectively. After storage at 4 °C for 1 or 3 days, ∼0.60 and ∼1.5 log loss of titer was measured (Fig. 1B). We set out to better understand the causes of rHCMV-1 instability and identify new candidate formulations to improve rHCMV-1 stability under these stress conditions.

### 3.2 Screening excipients for freeze-thaw stabilization of rHCMV-1

**Fig. 2:**
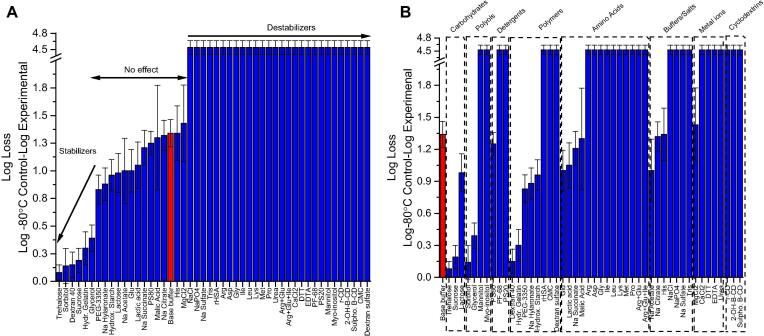
Log loss of rHCMV-1 viral infectivity titer after 3 freeze-thaw cycles from −80 °C in the presence of 48 different excipients. (A) Excipients listed in order of protective effect on viral vector stability, and (B) excipients listed by class of additives. The rHCMV-1 stock was diluted 1:150 into each excipient containing solution in a base buffer (10 mM Histidine, pH 6.5) in polypropylene microcentrifuge tubes, subjected to 3 freeze-thaw cycles and analyzed by IE-IFA. Log loss of viral titer was calculated by subtracting the log (titer) of the control sample, which was rHCMV-1 bulk diluted 1:150 in TNS buffer and stored at −80 °C, from the log(titer) of the experimental formulations after 3 freeze-thaw cycles. Stability data are an average of three measurements and the error bars represent the standard deviation. The viral vector in base buffer alone (10 mM Histidine, pH 6.5) is indicated by the red colored bar.

The ability of various classes and types of pharmaceutical excipients to stabilize the rHCMV-1 vector against three freeze-thaws (FT) was determined. A base buffer (10 mM Histidine, pH 6.5) alone was determined to be non-optimal since after undergoing three freeze-thaw cycles (−80 °C to room temperature), >1 log loss in rHCMV-1 vector titer was observed (red bar, Fig. 2). Forty-eight different excipients (individually prepared in base buffer) were evaluated for their ability to stabilize rHCMV-1 against FT stress (Fig. 2). Over half of the excipients were destabilizers resulting in complete rHCMV-1 titer loss, while 17 excipients showed either no effect or improved rHCMV-1 stability compared to rHCMV-1 in the base buffer alone (Fig. 2). Six additives (trehalose, sorbitol, dextran 40, sucrose, hydrolyzed gelatin, and glycerol) resulted in <0.5 log loss of viral titer after 3 FT cycles (Fig. 2A from most to least stable), and 11 excipients had no effect on rHCMV-1 vector titer loss given assay variability. The excipient categories that stabilized rHCMV-1 vs. FT stress included polysaccharides, polyols, and polymers (Fig. 2B). The majority of amino acids and salts resulted in complete rHCMV-1 titer loss during FT cycling, suggesting destabilization could be related to increased ionic strength of the formulation.

### 3.3 Screening excipients for 4 °C storage stabilization of rHCMV-1

**Fig. 3:**
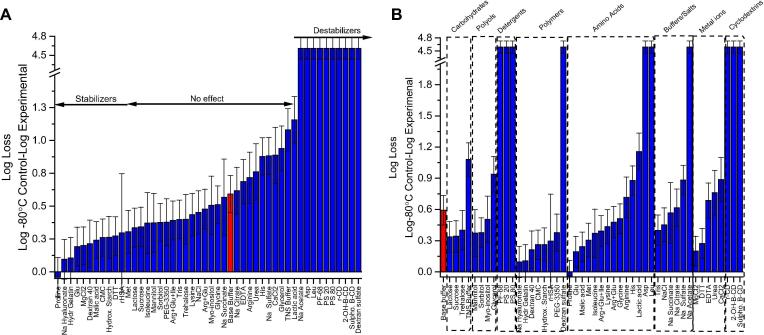
Log loss of rHCMV-1 infectivity titer after storage for 3 days at 4 °C in the presence of 48 different excipients. (A) Excipients listed in order of protective effect on viral vector stability, and (B) excipients listed by class of additives. The rHCMV-1 stock was diluted 1:150 into each excipient containing solution in a base buffer (20 mM sodium phosphate, 150 mM NaCl, pH 7.5) in polypropylene microcentrifuge tubes, subjected to incubation at 4 °C for 3 days, and analyzed by IE-IFA. Log loss of viral titer was calculated by subtracting the log (titer) of the control sample, which was rHCMV-1 bulk diluted 1:150 in TNS buffer and stored at −80 °C, from the log(titer) of the experimental formulations after storage at 4 °C for 3 days. Stability data are an average of three measurements and the error bars represent the standard deviation. The viral vector in base buffer alone (20 mM sodium phosphate, 150 mM NaCl, pH 7.5) is indicated by the red colored bar.

The same 48 excipients were evaluated for stabilizing effects on rHCMV-1 after incubation for 3 days at 4 °C. A different base buffer (20 mM sodium phosphate, 150 mM NaCl, pH 7.5) was utilized (which resulted in ∼0.7 log loss of rHCMV-1 infectivity titer vs. ∼0.4 log loss in 10 mM histidine, pH 6.5 base buffer). Approximately one quarter of the excipients were destabilizers resulting in higher rHCMV-1 titer losses, about half of the additives had no effect given assay variability, and about one quarter of compounds improved rHCMV-1 stability to varying extents compared to base buffer alone (Fig. 3). Promising excipients that resulted in ≤∼0.30 log loss of rHCMV-1 titer after storage for 3 days at 4 °C included proline, sodium hyaluronate, hydrolyzed gelatin, glutamic acid, MgCl_2_, dextran 40, malic acid, carboxymethyl cellulose, hydroxyethyl starch, DTT, rHSA, and methionine (Fig. 3A). Most of these additives are from one of the following excipient categories: polymers, organic compounds, and amino acids (Fig. 3B). Interestingly, hydrolyzed gelatin and dextran 40 were also identified as good freeze-thaw stabilizers (see above). Some, but not all, of these promising excipients are found in currently marketed viral vaccine formulations [26].

### 3.4 Excipient combinations to design candidate formulations of rHCMV-1

**Fig. 4:**
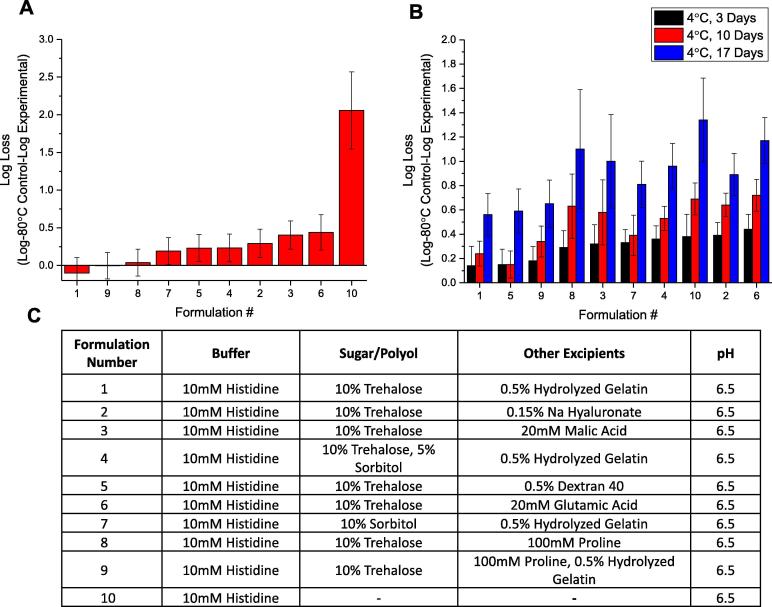
Stability profile of rHCMV-1 viral titer after multiple freeze-thaw cycles and after incubation at 4 °C (up to 17 days) in presence of various combinations of stabilizing excipients. (A) Log loss in rHCMV-1 titer after 5 freeze-thaw cycles from −80 °C, and (B) Log loss in rHCMV-1 titer after incubation at 4 °C for 3, 10 and 17 days. (C) Composition of various excipient combinations. The rHCMV-1 stock was diluted 1:100 into each formulation, stressed as indicated and viral titer was measured by IE-IFA. Log loss was calculated by subtracting the log (titer) of the control sample, which was rHCMV-1 bulk diluted 1:100 into TNS buffer and stored at −80 °C, from the log (titer) of the stressed experimental formulations. Stability data are an average of six measurements with the error bars representing the standard deviation.

After identification of stabilizing excipients from initial excipient screening studies (Fig. 2, Fig. 3), the “hits” were titrated in terms of concentration to identify the minimal amount required for a stabilizing effect on the rHCMV-1 vector (data not shown). We then combined stabilizing excipients from various classes to develop candidate formulations that minimized titer loss under more aggressive stress conditions including 5 freeze-thaw (FT) cycles and storage at 4 °C up to 17 days. For this set of experiments, a base buffer of 10 mM Histidine, pH 6.5 was selected since this value is the pH range optimal for vector stability (discussed below). After 5 FT cycles, rHCMV-1 titer loss was >2 log in base buffer alone (Fig. 4A). The rHCMV-1 titer losses in the excipient combination solutions after 5 FT cycles (graphed in order of decreasing effectiveness in Fig. 4A with composition listed in Fig. 4C) were greatly reduced and in some cases eliminated entirely (e.g., formulations 1, 8, and 9). Note, that only trehalose and sorbitol were evaluated as sugars/polyols due to reduced liquid stability of rHCMV-1 in sucrose containing formulations (data not shown). rHCMV-1 titer losses after 5 FT cycles were higher (0.40–0.44 log) in formulations containing 20 mM malic acid or 20 mM glutamic acid, suggesting these compounds are rHCMV-1 freeze-thaw destabilizers when combined with other additives. The rHCMV-1 titer loss after 5 freeze-thaw cycles in the remaining formulations was ∼0.25 log, which was largely within assay variability (Fig. 4A).

Next, the rHCMV-1 titer losses in the excipient combination solutions after incubation at 4 °C for up to 17 days (graphed in order of decreasing effectiveness in Fig. 4B with composition listed in Fig. 4C) were compared to the vector in base buffer. In this experiment, 4 °C samples were tested directly and not frozen prior to titration by IE-IFA assay. Formulations 1, 5, and 9 showed the best rHCMV-1 liquid stability profiles (Fig. 4B). These formulations all contained 10% (w/v) trehalose combined with either hydrolyzed gelatin (formulation 1), dextran 40 (formulation 5), or a combination of 100 mM proline with 0.5% (w/v) hydrolyzed gelatin (formulation 9) (Fig. 4B). Since rHCMV-1 titer losses were higher with proline (Formulation 9) than without proline (Formulation 1) in this combination formulations, there was no notable improvement in rHCMV-1 stability in formulations containing proline in the presence of hydrolyzed gelatin. Similarly, combinations that included sorbitol, malic acid, sodium hyaluronate, and glutamic acid resulted in higher levels rHCMV-1 titer losses after 17 days at 4 °C than formulations that contained either hydrolyzed gelatin or dextran 40 (Fig. 4B). These results provide additional evidence that polymers, specifically hydrolyzed gelatin and dextran 40, are good rHCMV-1 stabilizers in the liquid state and during freeze-thaw, especially in combination with trehalose.

**Fig. 5:**
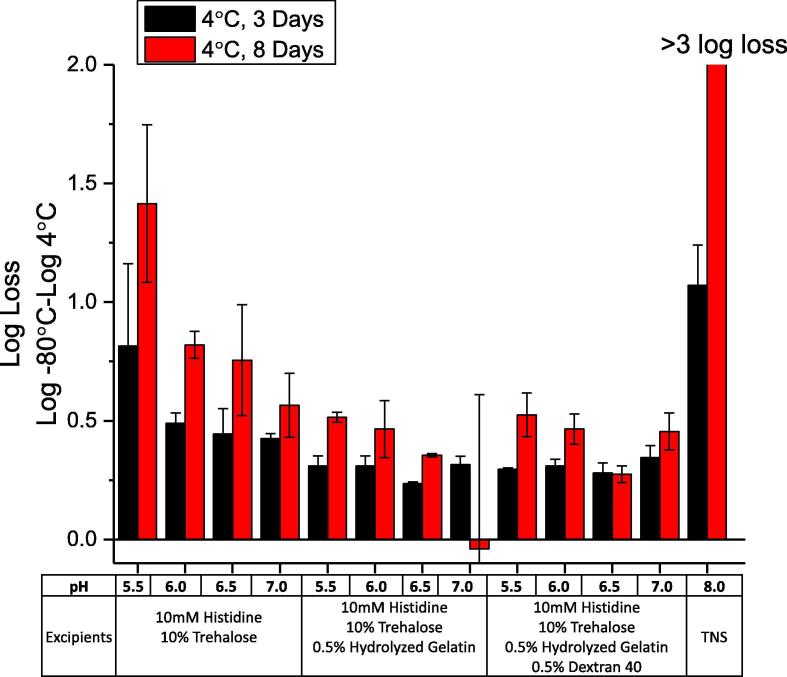
Effect of solution pH on log loss of rHCMV-1 infectivity titer after short term storage at 4 °C in the presence of various excipient combinations. The rHCMV-1 stock was diluted 1:100 into various formulations (10 mM histidine buffer at pH 5.5, 6.0, 6.5, and 7.0 in presence of the indicated excipients) and stored at 4 °C for up to 8 days. The stability of rHCMV-1 in TNS buffer was also examined. The log loss of rHCMV-1 viral titer was calculated by subtracting the log (titer) of the respective −80 °C control samples in the same formulation from the log (titer) of the experimental sample. Stability data are an average of six measurements with the error bars representing the standard deviation.

To determine if there was a solution pH effect on the rHCMV-1 liquid stability profile of in the presence of the top stabilizing excipients (i.e., trehalose, hydrolyzed gelatin, and dextran 40), the vector was incubated in various formulations at 4 °C for 3 and 8 days. The rHCMV-1 stock was diluted into a base buffer (10 mM histidine buffer, pH 5.5, 6.0, 6.5, 7.0) with three excipient combinations including trehalose, trehalose/hydrolyzed gelatin, and trehalose/ hydrolyzed gelatin/dextran 40 (Fig. 5), and results were compared to the vector in TNS buffer. In this experiment (and subsequent stability studies described below), each rHCMV-1 formulation (both stressed and non-stressed −80 °C control samples) underwent one freeze-thaw cycle prior to titer determinations by IE-IFA assay. As shown in Fig. 5, rHCMV-1 titer losses trended higher at pH 5.5 and 6.0 compared to the same formulations at pH 6.5 and 7.0. Vector titer losses in TNS buffer (pH 8.0) were also substantial. Taken together, these results indicate the optimal pH conditions for rHCMV-1 liquid stability as the pH range 6.5–7.0. Addition of 0.5% (w/v) hydrolyzed gelatin and/or 0.5% (w/v) dextran 40 reduced rHCMV-1 titer loss at pH 5.5 and 6.0, confirming these two excipients are good stabilizers of rHCMV-1 in liquid state, and they can each partially overcome vector destabilization under slightly acidic pH solution conditions. The rHCMV-1 vector titer losses were comparable in formulations that contained hydrolyzed gelatin and dextran 40 individually vs combined, thus there was no additive effect of using both polymers together to stabilize rHCMV-1 (Fig. 5).

### 3.5 Accelerated, real-time and freeze-thaw stability studies of rHCMV-1 in three candidate formulations

**Fig. 6:**
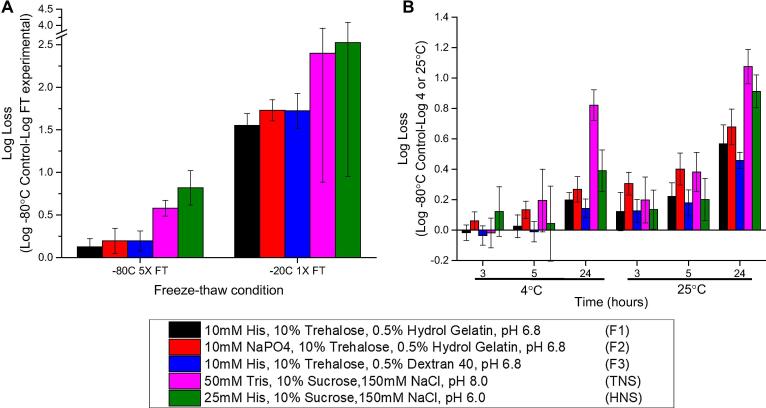
Stability profile of rHCMV-1 in three candidate formulations after multiple freeze-thaw cycles (at −20 and −80 °C) and short-term storage after thawing (at 4 and 25 °C) as compared to rHCMV-1 in TNS and HNS buffers. (A) Log loss in rHCMV-1 titer after 1 and 5 freeze-thaw cycles from either −20 °C or −80 °C. (B) Log loss in rHCMV-1 titer after 3, 5, and 24 h incubation at 4 and 25 °C. The rHCMV-1 stock was diluted 1:100 into each formulation, samples were stressed as indicated, and assayed by IE-FFA (stability samples were frozen at −80 °C prior to titration). The log loss was calculated by subtracting the log (titer) of the respective −80 °C control samples in the same formulations from the log (titer) of the experimental sample. Stability data are an average of four measurements and the error bars represent the standard deviation.

**Fig. 7:**
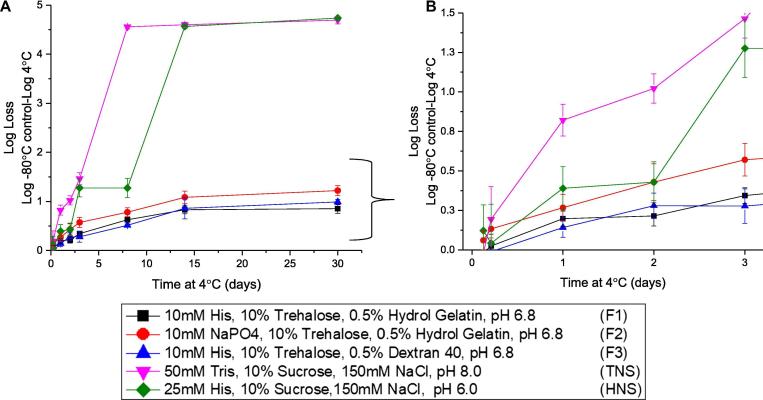
Stability profile of rHCMV-1 in three candidate formulations after storage at 4 °C for up to 30 days as compared to rHCMV-1 in TNS and HNS buffers. A) Log loss in rHCMV-1 titer as a function of storage time at 4 °C over 30 days, and (B) the stability data with axes enlarged through the first 3 days of incubation. The rHCMV-1 stock was diluted 1:100 into each formulation, samples were stressed as indicated, and assayed by IE-FFA (stability samples were frozen at −80 °C prior to titration). The log loss was calculated by subtracting the log (titer) of the respective −80 °C control samples in the same formulations from the log (titer) of the experimental sample. Stability data are an average of four measurements and the error bars represent the standard deviation.

Three candidate rHCMV-1 formulations were selected based on the studies described above and evaluated head-to-head in accelerated, real-time and freeze-thaw stability studies in pharmaceutical glass vials/stoppers as shown in Fig. 6, Fig. 7. The candidate formulations (F1-F3) each included a buffering agent at pH 6.8, a sugar and a polymer as follows: (F1) 10 mM histidine, 10% trehalose, 0.5% hydrolyzed gelatin, pH 6.8, (F2) 10 mM sodium phosphate, 10% trehalose, 0.5% hydrolyzed gelatin, pH 6.8, and (F3) 10 mM histidine, 10% trehalose, 0.5% dextran 40, pH 6.8. Both hydrolyzed gelatin and dextran 40 were evaluated as the polymeric additive since both consistently demonstrated improved rHCMV-1 stability in the liquid state and also provided some freeze-thaw stabilization. Inclusion of 10% (w/v) trehalose as the sugar was primarily based on its freeze-thaw stabilization properties, however, trehalose also outperformed sucrose and sorbitol as a stabilizer in the liquid state when formulated in 10 mM histidine, pH 6.5 buffer (data not shown). Sodium phosphate and histidine buffers were both selected to determine if there was an effect of buffer type on rHCMV-1 vector stability during freeze-thaw and 4 °C storage. The target pH of 6.8 was used since the pH range of 6.5 and 7.0 was shown to be optimal (Fig. 5). The stability of rHCMV-1 prepared in these three candidate formulations (F1-F3) were directly compared with the vector in TNS buffer (50 mM Tris, 150 mM NaCl, 10% (w/v) sucrose, pH 8.0) and HNS buffer (a formulation detailed elsewhere [27] containing 25 mM Histidine, 150 mM NaCl, 10% sucrose, pH 6.0).

We first evaluated the freeze-thaw stability of rHCMV-1 in the five formulations at both −20 °C and −80 °C, since −20 °C freezer storage is more widely available in most clinical settings. Short-term liquid stability of rHCMV-1 was also evaluated for 1 day at 4 and 25 °C. In the three candidate formulations, a 0.13–0.20 log loss in titer after 5 freeze-thaw cycles at −80 °C was observed. In contrast, rHCMV-1 in TNS and HNS buffers lost 0.58 and 0.82 log titer after 5 FT cycles (Fig. 6A). One probable reason for decreased rHCMV-1 stability in TNS and HNS buffers during FT is the presence of 150 mM NaCl, which was shown to greatly increase titer loss after FT during excipient screening studies (Fig. 2). The rHCMV-1 titer losses due to freeze-thaw from storage at −20 °C were much higher than at −80 °C. For example, after a single freeze-thaw cycle, rHCMV-1 in the three candidate formulations lost 1.6–1.7 log of viral infectivity titer while the vector in HNS and TNS formulations lost ∼2.5 log titer (Fig. 6A). Taken together, the three candidate formulations outperformed the HNS and TNS control formulations under all freeze-thaw stress conditions examined (Fig. 6A), albeit rHCMV-1 is relatively unstable due to a single freeze-thaw from storage at −20 °C under all examined conditions.

Similar liquid state storage stability profiles of rHCMV-1 in the five formulations were observed after 5 h at 4 and 25 °C (given assay variability), although the formulations with histidine displayed a trend of improved stability at the same pH value (Fig. 6B). After 24-hour incubation, rHCMV-1 showed better stability in the three candidate formulations compared to TNS and HNS buffers at both temperatures (Fig. 6B). Similarly, over the course of 30 days at 4 °C, rHCMV-1 was found to be most stable in the three candidate formulations compared to TNS and HNS buffers (Fig. 7A and B). After a 3 day incubation at 4 °C, rHCMV-1 titer loss in TNS and HNS control formulations was > 1 log, whereas in the best candidate formulations, rHCMV-1 titer loss was ∼0.25 log (Fig. 7B). Comparatively, rHCMV-1 was not as stable at 4 °C in candidate formulations with sodium phosphate vs histidine buffer (Fig. 7B). After one month at 4 °C, the rate of degradation of rHCMV-1 in the control TNS/HNS formulation buffers was significantly higher compared to the three candidate formulations. For example, in TNS and HNS buffers, rHCMV-1 lost ∼1 log of titer after 3 days at 4 °C, while in the three candidate formulations, rHCMV-1 lost 0.85, 1.22, and 0.99 titer after 30 days 4 °C (Fig. 7A).

## 4 Discussion

There is growing interest in developing live, attenuated viruses not only as vaccine candidates themselves, but as engineered vectors for vaccination against other viral diseases and as “live drugs” including therapeutic oncolytic agents. For example, engineered versions of herpes simplex virus, vesicular stomatitis virus, Newcastle disease virus, adenovirus, and poxvirus haven been used in oncolytic virotherapy [28], [29]. In addition, a live, adenoviral associated virus vector based drug, LUXTURNA™ was recently approved for treatment of retinal dystrophy [30]. There is particular interest to evaluate recombinant CMV vectors for HIV vaccination based on their ability to protect against SIV in non-human primates, as well as to facilitate clearance of this highly virulent virus [5], [9], [31]. Some RhCMV-based vectors were shown to not only induce high levels of effector memory T-cells but to elicit CD8+ T-cells that recognize target antigens presented by MHC-II and MHC-E instead of classical MHC-I [32], [33]. Recombinant CMV based vectors thus represent a unique vaccine platform that can be genetically programmed to elicit a diverse set of T cell responses to inserted antigens of important infectious diseases including HIV-1, tuberculosis, and malaria [4], [6], [8]. Growing interest in clinical applications of viral vectors has led to the need to address instability issues during manufacturing, storage and patient administration. For example, IMLYGICTM, a live, recombinant HSV for treatment of melanomas, is stored frozen at ≤−70°, and is stable for up to six weeks after thawing and subsequent storage at 2–8 °C [34]. LUXTURNA™ is also stored as a frozen liquid formulation at ≤−65 °C [30]. Although, ultralow frozen storage temperatures are possible for biological drugs/vaccines administered in an inpatient setting and for early clinical trials, such storage conditions are largely impractical for widespread vaccination programs especially in the developing world [14].

We did not examine the effect of gene deletions and antigen insertion on the freeze-thaw and storage stability of rHCMV-1. Previous studies examining the thermal stability profiles of various CMV strains reported that virus stability varied by strain type, buffer composition, and harvest time during viral replication [35]. While we cannot rule out that the deletion of virion proteins such as subunits of the pentameric complex or phosphoprotein 71 might impact stability, it seems unlikely that these genetic modifications impact the biophysical product characteristics since these are not major structural components involved in the formation and integrity of the viral capsid, tegument or envelope. As such, we consider it highly likely that the results obtained in this study are generally applicable to HCMV.

With respect to improving freeze-thaw (FT) stability of rHCMV-1, although several compounds were identified that provide modest to good rHCMV-1 stability enhancements, 10% (w/v) of sucrose, sorbitol, or trehalose best protected rHCMV-1 against infectivity titer loss after 3 FT cycles at −80 °C. These results are consistent with previous reports of compounds that can presumably stabilize the enveloped virus particle’s structural integrity during freezing by maintaining osmotic equilibrium across the lipid bilayer [18], [36], [37]. In addition, sugars and polyols are well known cryoprotectants for vaccines and proteins based drugs [14], [38]. Nevertheless, these promising stabilizers could not fully protect rHCMV-1 from inactivation during even one FT cycle at −20 °C, likely due to incomplete freezing of the solution in the presence of these cryoprotectants (since these additives have glass transition temperatures below this temperature), a condition known to lead to changes/shifts in solution pH, ionic strength and excipient concentrations [14], [15]. We also observed that addition of NaCl (and other salts) led to increased rHCMV-1 titer losses during FT. This observation suggests that the total ionic strength of the formulation is an important variable in the FT stability of rHCMV-1, and NaCl as well as other ionic additives should be avoided. Interestingly, with an unrelated live Lentiviral vector, we recently observed a link between ionic strength of the formulation and physical adsorption of the vector to the primary container [39]. Nonetheless, other viruses display different stability profiles when ionic strength is varied demonstrating unique aspects of the stability profile of each live viral vaccine/vector candidate [40], [41], [42]. For example, aggregation of the HSV-1 virions contributes to viral infectivity losses due to freeze-thaw in the absence of cryoprotectants [29]. Mechanistically, freeze-thaw mediated inactivation may result not only from viral particle aggregation, but also osmotic shock [36]. Further experiments are planned to gain a better understanding of the causes and mechanisms of rHCMV-1 vector instability vs. ionic strength of solution, especially during freezing and thawing.

For improving liquid storage stability of rHCMV-1, optimization of solution pH played a major role. Solutions at pH 5.5, 6.0, and 8.0 were less stable compared to pH 6.5 and 7.0, and pH 6.8 was selected as the optimal solution pH for vector stability. Histidine buffer provided improved liquid stability of rHCMV-1 compared to sodium phosphate and Tris, which was especially evident during short-term (i.e., 5 h) incubation at 25 °C (Fig. 6). Although sucrose, trehalose, and sorbitol were all effective freeze-thaw stabilizers, trehalose outperformed sucrose and sorbitol in slowing the rate of degradation of rHCMV-1 in the liquid state. Polymers such as hydrolyzed gelatin, dextran 40, and sodium hyaluronate improved the vector’s stability in liquid formulations. Interestingly, the stabilizing effects of these polymers were not additive, while the effective stabilizing concentrations of sodium hyaluronate increased the solution viscosity to impractical levels (∼6 cP).

In summary, the rate of rHCMV-1 inactivation in the liquid state differed quite notably based on the excipient composition of the formulation. For example, in TNS buffer, rHCMV-1 loses >1 and >4 log titer after 3 and 8 days at 4 °C, respectively. In comparison, in a candidate formulation of 10 mM histidine, 10% trehalose, 0.5% hydrolyzed gelatin, pH 6.8, rHCMV-1 infectivity titer losses were much less under the same conditions (∼0.3 and ∼0.6 log). In non-human primates a ∼0.5 log difference in titer of HCMV-based vectors resulted in the ability or inability of a given vector to elicit T cell responses to the antigen insert [21]. Thus, a ∼0.5 log loss in titer might represent the difference between success and failure to eliciting an immune response in humans. These observations highlight the importance of maintaining the infectivity titer values of the viral vector product candidate within a known range throughout manufacturing, clinical handling and patient administration.

Hydrolyzed gelatin and dextran 40 were the most effective stabilizers identified in this study at reducing the rate of rHCMV-1 titer loss when stored in the liquid state at 4 °C. Hydrolyzed gelatin is used in many lyophilized commercial live viral vaccines including MMR-II®, ProQuad®, Rabavert®, Zostavax®, and Varivax® [26]. Nonetheless, liquid stability is still an important consideration to retain viral infectivity titers during manufacturing (i.e., formulation and filling) as well as after reconstitution during the day of administration to patients. Although the precise mechanism of hydrolyzed gelatin mediated stabilization is poorly understood, it has been hypothesized that hydrolyzed gelatin non-specifically binds and stabilizes the virions [15], [43]. Since hydrolyzed gelatin is an animal-derived excipient, there is ongoing interest in identifying alternative polymeric stabilizers for live virus vaccine/vectors. Presumably dextran 40 mediated stabilization occurs by similar mechanisms, yet dextran 40 is not commonly used in live virus formulations [26]. Recombinant human gelatin has been reported to be a suitable substitute for hydrolyzed porcine gelatin in a refrigerator stable live Varicella vaccine, since both lyophilized vaccine formulations showed similar profiles of viral infectively loss over 24 months at 2–8 °C [43]. Moreover, another study found that a low molecular weight recombinant human gelatin was superior to its animal derived counterpart [44], although commercial sourcing of a recombinant replacement is challenging. A recently study showed the addition of urea in the presence of hydrolyzed gelatin enabled a refrigerator stabile lyophilized formulation of Varicella and ProQuad® (measles, mumps, rubella, varicella) vaccines [45], [46].

Despite identifying stabilizing excipient combinations and optimized solution conditions for storage as a frozen liquid at −80 °C, the storage stability limitations of rHCMV-1 in a 2–8 °C liquid formulation and as a frozen liquid at −20 °C were noted. Due to the inherent instability of rHCMV-1 and the target geographical location where this vaccine candidate will be administered in the developing world (sub-tropical and tropical climates), one can foresee that a more stable vaccine drug product is necessary for successful wide-spread vaccination programs, since ultralow temperature storage (i.e., ≤−70 °C) is often impractical at a commercial scale [47], and maintaining an ultralow temperature frozen vaccine cold chain in the developing world would be challenging [14], [48]. Although a frozen liquid formulation of rHCMV-1 can facilitate more rapid initiation of early clinical trials performed under more controlled conditions of storage, transport and administration, the development of lyophilized formulations appears to be the next step in formulation development to maintain rHCMV-1 vaccine potency during long-term storage and distribution as a commercial vaccine dosage form especially for use in the developing world.

## Declaration of Competing Interest

The authors declare the following financial interests/personal relationships which may be considered as potential competing interests: OHSU and PC, GD, WP, and KF have financial interests in VirBio Technology, Inc., a company that may have a commercial interest in the results of this research and technology. This potential conflict of interest has been reviewed and managed by OHSU.
